# Supplementary material for: Mitochondrial electron transport chain is necessary for NLRP3 inflammasome activation
Source: Nat Immunol. 2022 Apr 28;23(5):692–704. doi: 10.1038/s41590-022-01185-3 (PMC9098388; doi:10.1038/s41590-022-01185-3)
Supplement: Supplementary file 1 — Reporting Summary [file 41590_2022_1185_MOESM1_ESM.pdf]

nature portfolio

Corresponding author(s): Navdeep Chandel

Last updated by author(s): 1/19/2022

Reporting Summary

Nature Portfolio wishes to improve the reproducibility of the work that we publish. This form provides structure for consistency and transparency in reporting. For further information on Nature Portfolio policies, see our Editorial Policies and the Editorial Policy Checklist.

Statistics

For all statistical analyses, confirm that the following items are present in the figure legend, table legend, main text, or Methods section.

n/a	Confirmed	
☐	☒	The exact sample size (*n*) for each experimental group/condition, given as a discrete number and unit of measurement
☐	☒	A statement on whether measurements were taken from distinct samples or whether the same sample was measured repeatedly
☐	☒	The statistical test(s) used AND whether they are one- or two-sided<br>*Only common tests should be described solely by name; describe more complex techniques in the Methods section.*
☐	☒	A description of all covariates tested
☐	☒	A description of any assumptions or corrections, such as tests of normality and adjustment for multiple comparisons
☐	☒	A full description of the statistical parameters including central tendency (e.g. means) or other basic estimates (e.g. regression coefficient) AND variation (e.g. standard deviation) or associated estimates of uncertainty (e.g. confidence intervals)
☐	☒	For null hypothesis testing, the test statistic (e.g. *F*, *t*, *r*) with confidence intervals, effect sizes, degrees of freedom and *P* value noted<br>*Give P values as exact values whenever suitable.*
☒	☐	For Bayesian analysis, information on the choice of priors and Markov chain Monte Carlo settings
☒	☐	For hierarchical and complex designs, identification of the appropriate level for tests and full reporting of outcomes
☒	☐	Estimates of effect sizes (e.g. Cohen's *d*, Pearson's *r*), indicating how they were calculated

Our web collection on statistics for biologists contains articles on many of the points above.

Software and code

Policy information about availability of computer code

Data collection	Oxygen consumption data was collected using Wave 2.4 software. Flow cytometry data was collected using FACS DIVA 8.0.3 software. Metabolite data was collected using Xclibur 4.1 software. Immunoblot data were collected using a Wes by ProteinSimple using Compass for SW software 5.0.1. RTPCR data was collected using CFX Manager (version 3.1) by Bio-Rad. RNASeq data was collected using Illumina NextSeq 500 system Raw BCL read files were demultiplexed and converted to FASTQ files using bcl2fastq (Illumina) and trimmed using Trimmomatic (version 0.39). Fluorescence data from the H2O2 data and ATP assay and colorometric data from BCA and ELISA were collected using SpectraMax M2 (Molecular Devices) and SoftMax Pro (Version 6.4). For metabolomics, high-resolution HPLC–tandem mass spectrometry was performed on a Q-Exactive (ThermoFisher Scientific) in line with an electrospray source and an UltiMate 3000 (ThermoFisher Scientific) and data were collected using Xcalibur 4.1 software.
Data analysis	GraphPad Prism 9.0 and MetaboAnalyst 4.0 were used for statistical tests. RNASeq data was analyzed using the R package edgeR6. Metabolite data was analyzed using Tracefinder 4.1 software. Immunoblot data were analyzed using Compass for SW software 5.0.1 (ProteinSimple). Flow cytometry data was analyzed using Flowjo 10.4.2. For RNASeq, reads were then aligned to the mouse mm10 reference genome using STAR to generate BAM files3, HTSeq was used to count reads in the exons of genes, and likelihood ratio tests for all samples and all detected transcripts and pairwise differential gene expression analyses were carried out using the R package DESeq2.

For manuscripts utilizing custom algorithms or software that are central to the research but not yet described in published literature, software must be made available to editors and reviewers. We strongly encourage code deposition in a community repository (e.g. GitHub). See the Nature Portfolio guidelines for submitting code & software for further information.

Data

Policy information about availability of data

All manuscripts must include a data availability statement. This statement should provide the following information, where applicable:

- Accession codes, unique identifiers, or web links for publicly available datasets
- A description of any restrictions on data availability
- For clinical datasets or third party data, please ensure that the statement adheres to our policy

All data from the manuscript are available from the corresponding author on request. Source data are provided with this paper. Rdata related to this paper is available on the GEO repository (accession number GSE197606)

Field-specific reporting

Please select the one below that is the best fit for your research. If you are not sure, read the appropriate sections before making your selection.

☒ Life sciences ☐ Behavioural & social sciences ☐ Ecological, evolutionary & environmental sciences

For a reference copy of the document with all sections, see nature.com/documents/nr-reporting-summary-flat.pdf

Life sciences study design

All studies must disclose on these points even when the disclosure is negative.

Sample size	All experiments were performed using sample sizes based on standard protocols in the field. We made every effort to avoid excessive or needless use of animals. No statistical tests were used to predetermine sample sizes. We used sample sizes commonly used in literature in the field (Weinberg 2019, Mills 2016, Coll 2015). We used statistical analysis consistent with the sample size for each experiment and found sufficient statistical power with the sample sizes used in our study.
Data exclusions	Outliers were determined using the ROUT method, Q = 1%. Experiments were excluded from analysis if the controls did not work; data from successfully completed experiments were not excluded unless they were determined to be outliers.
Replication	All experimental data were reliable reproduced in multiple independent experiments as indicated in the figure legends. For in vivo experiments, multiple mice were used in at least two independent cohorts to ensure reproducibility.
Randomization	Transgenic mice were predetermined by mouse genotype and therefore could not be randomized. C57Bl/6J mice were randomly assigned to treatment and control groups. All mice were age-matched and littermates.
Blinding	Investigators were not blinded. Blinding was not possible as predominately one person was responsible for performing each experiment and carrying out data analysis.

Reporting for specific materials, systems and methods

We require information from authors about some types of materials, experimental systems and methods used in many studies. Here, indicate whether each material, system or method listed is relevant to your study. If you are not sure if a list item applies to your research, read the appropriate section before selecting a response.

Materials & experimental systems

n/a	Involved in the study	
☐	☒	Antibodies
☒	☐	Eukaryotic cell lines
☒	☐	Palaeontology and archaeology
☐	☒	Animals and other organisms
☒	☐	Human research participants
☒	☐	Clinical data
☒	☐	Dual use research of concern

Methods

n/a	Involved in the study	
☒	☐	ChIP-seq
☐	☒	Flow cytometry
☒	☐	MRI-based neuroimaging

Antibodies

Antibodies used	Antibodies used for immunoblot: anti-Vinculin (Cell Signaling, #13901, clone E1E9V; 1:500 dilution); anti -IL-1beta (R&D systems, #AF-401-NA; 1:200 dilution); anti-caspase-1 (p20)(Adipogen, AG-20B-0042-C100, clone Casper-1; 1:250 dilution); anti-ASC (Novus Biologics, NBP1-78977SS; 1:50 dilution); anti-NLRP3 (Novus Biologics, NBP2-03948SS, clone 25N10E9; 1:100 dilution)
Validation	The antibodies used in this study were tested by the manufacturer

Validation

-anti-Vinculin (Cell Signaling, #13901, clone E1E9V). This antibody can be found in 85 citations. The manufacturer also provides antibody testing data: https://www.cellsignal.com/products/primary-antibodies/vinculin-e1e9v-xp-rabbit-mab/13901.

-anti-IL-1beta (R&D systems, #AF-401-NA). This antibody can be found in 150 citations. The manufacturer also provides antibody testing data: https://www.rndsystems.com/products/mouse-il-1beta-il-1f2-antibody_af-401-na

-anti-Caspase-1 (p[illegible](clone Casper-1, Adipogen; catalog number AG-20B-0042-C100). This antibody can be found in 28 citations. The manufacturer also provides antibody testing data: https://adipogen.com/ag-20b-0042-anti-caspase-1-p20-mouse-mab-casper-1.html

-anti-ASC (Novus Biologics, NBP1-78977SS). This antibody can be found in 26 publications. The manufactucurer also provides antibody testing data: https://www.novusbio.com/products/asc-tms1-antibody_nbp1-78977#supportresearch

-anti-NLRP3 (Nobus Biologics, NBP2-03948SS, clone 25N10E9). The antibody can be found in 1 publication. The manufactuer also provides antibody testing data (https://www.novusbio.com/products/nlrp3-nalp3-antibody-25n10e9_nbp2-03948#protocols-faqs)

Animals and other organisms

Policy information about studies involving animals; ARRIVE guidelines recommended for reporting animal research

Laboratory animals

Both male and female C57Bl/6J mice were used. Mice were 8-14 weeks old. Rosa26NDI1-lsl/wt and Rosa26AOX-lsl/wt mice were mixed C57Bl/6 J/N. Rosa26NDI1-lsl/wt mice were genotyped using the following primers: Rosa26 Fwd 5’ – GAGTTCTCTGCTGCCTCCTG; Rosa26 Rev 5’ – CCGACAAAACCGAAAATCTG; and WPRE B Fwd 5’ – GACGAGTCGGATCTCCCTTT. Rosa26AOX-lsl/wt mice were genotyped using the following primers: AOX lsl Fwd 5’-GCGATGCAAGATGGAGGGTA-3’; AOX lsl Rev 5’-TGAATCCAACCGTGGTCTCG-3’; Rosa26 Fwd 5’-GACCTCCATCGCGCACTCCG-3; and Rosa26 Rev 5 -CTCCGAGGCGGATCACAAGC-3. VAV-iCre mice were genotyped using the following primers: Fwd 5’-AGATGCCAGGACATCAGGAACCTG-3’ and Rev 5’ ATCAGCCACACCAGACACAGAGATC-3’. QPC floxed and wildtype allelles were genotyped using the following primers: QPC Fwd 5’-CTTCCGCTCCTCCCGGAAGT; QPC Rev 5’- TTCCCAAACTCGCGGCCCATG. LysM mice were genotyped using the following primers: LysM-Cre 5’- CRECCCAGAAATGCCAGATTACG; LysM-Pro 5’- GCATTGCAGACTAGCTAAAGGCAG; LysM ex-1 5’- GTCCGCCAGGCGGACTCCATAG

Wild animals

This study did not involve wild animals

Field-collected samples

This study did not involve samples collected from the field.

Ethics oversight

All mouse work was done in accordance with Northwestern University Institutional Animal Care and Use Committee (IACUC).

Note that full information on the approval of the study protocol must also be provided in the manuscript.

Flow Cytometry

Plots

Confirm that:

☒ The axis labels state the marker and fluorochrome used (e.g. CD4-FITC).

☒ The axis scales are clearly visible. Include numbers along axes only for bottom left plot of group (a 'group' is an analysis of identical markers).

☒ All plots are contour plots with outliers or pseudocolor plots.

☒ A numerical value for number of cells or percentage (with statistics) is provided.

Methodology

Sample preparation

Sample preparation is described in detail in the methods section of the manuscript.

BMDMs were plated at 2 million cells/well in a 12-well plate and allowed to adhere overnight. TMRE was added at a concentration of 200nM for 30 minutes. Cells were washed with PBS and removed from the plate with Accutase before resuspension in PBS supplemented with 10% NU-Serum IV.

Instrument

BD FACSymphony A5-Laser Anaylzer

Software

BD FASC Diva was used for collection of the data. All data was analyzed using FlowJo software.

Cell population abundance

Cells were not sorted

Gating strategy

Example gating strategy is provided in Ext Fig 6. FSC-A vs SSC-A was used to determine cell populations from debris. From this population, FSC-A vs FSC-H was used to determine single cells. Geometric mean of TMRE was used to determine relative MFI of treated samples compared to untreated samples.

☒ Tick this box to confirm that a figure exemplifying the gating strategy is provided in the Supplementary Information.
